# Motivation for Sustained Substance Use in Patients with Psychotic Disorders

**DOI:** 10.1192/j.eurpsy.2025.998

**Published:** 2025-08-26

**Authors:** C. Tapoi

**Affiliations:** 1 Addictions Department, Alexandru Obregia Clinical Psychiatry Hospital, Bucharest, Romania

## Abstract

**Introduction:**

Substance use is a prevalent issue in individuals with psychotic disorders and has major implications for the course of the disease. Substance use is associated with treatment noncompliance, more positive symptoms and increased risk of relapse. However, reasons for substance use in people diagnosed with psychotic disorders are insufficiently understood.

**Objectives:**

This study seeks to explore the specific reasons for substance use among patients diagnosed with psychotic

disorder, while aiming to identify subgroups of patients that may benefit from targeted interventions to reduce drug use.

**Methods:**

We investigated the reasons for maintaining substance use in patients with a dual diagnosis of psychotic disorder and substance use disorder that were admitted to Alexandru Obregia Clinical Psychiatric Hospital, Addictions Department, Bucharest, Romania, between October 2024 and March 2025. Patients were evaluated through a semi-structured interview developed for this study.

**Results:**

The results of the study will be available and presented during the EPA 2025 Congress.

**Conclusions:**

Understanding the underlying motivations for substance use is crucial for developing targeted interventions that address the unique needs of patients with psychotic disorders, ultimately improving treatment outcomes and promoting long-term recovery.

**Disclosure of Interest:**

None Declared

